# Bridging strategies for heart-lung transplantation: Current concepts and mechanical support modalities

**DOI:** 10.1016/j.jhlto.2026.100589

**Published:** 2026-05-07

**Authors:** K. Hoetzenecker, J.W. Stokes, M. Bacchetta, O. Mercier

**Affiliations:** a Vanderbilt University Medical Center, Department of Thoracic Surgery, Nashville, TN; b Vanderbilt University Medical Center, Department of Cardiac Surgery, Nashville, TN; c Department of Biomedical Engineering, Vanderbilt University, Nashville, TN; d Department of Thoracic Surgery and Heart-Lung Transplantation, Université Paris-Saclay, Hôpital Marie Lannelongue, Le Plessis Robinson, France; e INSERM UMR_S 1358, HPPIT, Hôpital Marie Lannelongue, FHU André Cournand, Le Plessis robinson, France

**Keywords:** Bridging, Heart-lung transplantation, ECMO, Sports-mode ECMO, Eisenmenger

## Abstract

Heart-lung transplantation (HLTx) has become increasingly rare over the past decades. However, HLTx remains the only life-saving option for patients experiencing end-stage cardiopulmonary failure. In parallel, extracorporeal membrane oxygenation (ECMO) has emerged as an established bridging strategy to isolated heart or lung transplantation. The role of ECMO before HLTx remains poorly defined, with current evidence limited to small series and case reports. Bridging patients to HLTx is complex, as support must address both gas exchange and hemodynamic instability in a highly heterogeneous population. The choice and configuration of ECMO must therefore be personnalized, guided by the patient’s underlying disease and the expected duration of support.

## Background

Recent literature on heart-lung transplantation (HLTx) is limited, as the practice of combined HLTx has become increasingly rare.^1^ This is mainly due to significant improvements in isolated heart and lung transplantation techniques, alongside the development of durable mechanical circulatory support systems and an improved understanding of right ventricle reverse remodeling in pulmonary vascular disease, which have significantly reduced the number of candidates requiring dual-organ replacement. Nonetheless, a subset of patients with end-stage cardiopulmonary disease continues to depend on HLTx as the only viable life-saving therapy.

Over the past decade, extracorporeal life support and extracorporeal membrane oxygenation (ECMO) have become accepted tools for bridging deteriorating patients to isolated lung and heart transplantations.^2^, ^3^ However, the extension of these strategies to heart-lung transplant candidates remain poorly defined, and the existing evidence is largely anecdotal or derived from small case series. The complexity of bridging HLTx candidates arises from the need to support not only gas exchange but also hemodynamic stability and a significant heterogeneity in underlying diseases and pathophysiology.

Patients listed for HLTx often present with advanced biventricular dysfunction, pulmonary hypertension, or complex congenital heart disease. The decision to initiate extracorporeal life support support, as well as the optimal configuration of ECMO or mechanical assist devices, must be individualized and guided by both the patient’s physiology and the anticipated duration of support. Bridging HLTx candidates follows the same principles outlined the recent ISHLT consensus for lung transplant candidates. These include considerations of multidisciplinary team structure, as well as relevant risk factors and contraindications.^4^

One of the main indications for combined HLTx is the coexistence of end-stage pulmonary disease—for example, interstitial lung disease (ILD), chronic obstructive pulmonary disease, or pulmonary arterial hypertension—together with irreversible cardiac dysfunction. In patients with group III pulmonary hypertension secondary to chronic lung disease, right ventricular (RV) dysfunction is often reversible following isolated lung transplantation. However, when left-sided cardiac disease coexists—such as restrictive cardiomyopathy, ischemic cardiomyopathy, or complex combined valvular pathology—the degree of ventricular impairment may preclude isolated LTx, necessitating a combined HLTx approach. When hemodynamic deterioration occurs while awaiting transplantation, temporary mechanical support can become necessary. The primary goal in this setting is to stabilize circulation, maintain end-organ perfusion, optimize gas exchange, and allow time for donor matching. Several ECMO configurations can be employed depending on the predominant physiological need.

## Femoro-femoral VA-ECMO

Veno-arterial ECMO (VA-ECMO) via bilateral femoral cannulation is the most accessible and a rapidly deployable form of support in a HLTx setting.^5^, ^6^ It can be initiated percutaneously at the bedside and provides both respiratory and circulatory support. Notably, VA-ECMO unloads the right ventricle but can increase left ventricle afterload.

Despite the above-mentioned advantages, a femoro-femoral VA-ECMO configuration is associated with important drawbacks. Retrograde flow through the descending aorta may result in differential hypoxemia—a condition in which the upper body and coronary circulation receive poorly oxygenated blood from the failing native heart, while the lower body receives oxygenated ECMO blood. This so called Harlequin Syndrome or North/South Syndrome can limit the use of a sole fem-fem VA-ECMO in patients with combined heart and lung failure. Additionally, bilateral femoral cannulation restricts mobilization and can make ambulation more challenging.^7^ This is an important limitation since muscle loss and deconditioning due to immobilization significantly increases perioperative risk.

For these reasons, femoro-femoral VA-ECMO is often considered a temporary rescue modality, a short-term bridging solution, or a configuration to stabilize a patient to more durable configurations.

## VAV-ECMO

A veno-arterial-venous (VAV) configuration addresses some limitations of a fem-fem VA-ECMO. By adding a return cannula to the right internal jugular or subclavian vein, VAV-ECMO equalizes oxygen delivery throughout the body and provides hemodynamic and respiratory stabilization in a single circuit.^8^

VAV-ECMO is particularly useful for patients with end-stage lung disease and secondary cardiac failure, as it allows partial unloading of the right ventricle while maintaining adequate oxygenation to the upper body and coronary arteries. The main drawback is its limitation in flow, since a single venous drainage cannula must entertain 2 return cannulas. Similarly, to a femoro-femoral VA-ECMO mobilization is complicated by the 2 groin cannulas.

## V-PA ECMO

Veno-pulmonary arterial ECMO (V-PA) or oxy-RVADs are a relatively new concept of extracorporeal support for patients with impaired gas exchange and RV dysfunction, and they have been successfully used in a bridge to LTx setting and in ARDS patients.^9^, ^10^ V-PA facilitates effective decompression of the right heart in patients with acute decompensation while also providing consistent and effective gas exchange by minimizing recirculation. Current evidence of V-PA ECMO in bridging patients to HLTx is limited to case reports.^11^, ^12^ At this current time, addition data is needed to determine the usefulness of V-PA ECMO in this setting and is currently not re--ed by the authors as a standard bridging concept.

## Sports-mode ECMO

**Figure 1:**
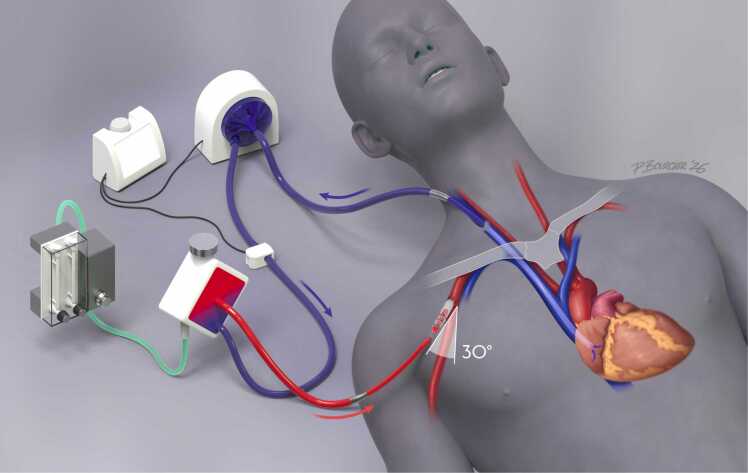
“Sports-mode” ECMO configuration involves surgical graft implantation into the subclavian artery for arterial return, combined with venous drainage via the internal jugular vein. The arterial graft should be implanted at a 30° angle to direct the ECMO flow towards the aortic arch and thus avoid hyperperfusion of the right arm.

The so-called “sports-mode” ECMO configuration represents a more physiologic and mobilization-friendly approach to long-term bridging for combined respiratory and hemodynamic support.^13^ This technique involves surgical graft implantation into the subclavian artery for arterial return, combined with venous drainage via the internal jugular vein. It allows the patient to remain fully ambulatory (Figure 1). Although technically more complex and requiring a surgical procedure, sports-mode ECMO is an optimal strategy for stable candidates with longer anticipated waiting times.^14^

By restoring antegrade arterial flow, sports-mode ECMO reduces the risk of differential hypoxemia to the brain. However, careful monitoring of coronary oxygenation is required, as native cardiac output may still deliver partially desaturated blood to the coronaries. Supplemental oxygen via nasal cannula or non-rebreather mask is commonly used to mitigate this effect. If the axillary artery is not accessible, cannulation of the ascending aorta through a small anterior thoracotomy in the third intercostal space can be considered.^15^

## Bridging to HLTx in severe left ventricular failure

In patients with severe left ventricular (LV) dysfunction, the initiation of VA-ECMO may inadvertently increase afterload, leading to LV distention, elevated end-diastolic pressures, and pulmonary congestion. These pathophysiological changes can reduce coronary perfusion pressure and exacerbate myocardial ischemia, potentially compromising candidacy for transplantation. With preserved LV function, the native heart may generate sufficient contractile force to overcome increased afterload; however, in severely depressed LV function, ejection is impaired, resulting in stagnant flow and thrombotic risk within the LV and aortic root. Thus, ventricular decompression becomes a key consideration in these patients.

Strategies for LV decompression include atrial septostomy, intra-aortic balloon pump, percutaneous transaortic LV assist devices, and direct LV venting. Each method carries unique risks, including vascular injury, hemolysis, or device thrombosis, and the optimal approach remains debated in the setting of bridging to HLTx.^16^, ^17^ In certain cases, indirect unloading via pulmonary artery drainage may offer sufficient decompression while avoiding the invasiveness of direct LV venting.^18^ Temporary BiVAD systems inserted in a minimal invasive way, such as the CentriMag ECMO-VAD hybrid (EC-VAD), are an alternative approach.^19^ They allow flexible configuration for right and left heart unloading while maintaining extracorporeal oxygenation if needed.

Ultimately, the strategy should be tailored to patient stability, anticipated bridging duration, and institutional expertise.

## Bridging patients with structural heart defects: Eisenmenger physiology

Patients with Eisenmenger syndrome, resulting from uncorrected atrial or ventricular septal defects (ASD or VSD) leading to chronic right-to-left shunting and pulmonary vascular remodeling, represent a particularly challenging bridging cohort. Understanding the size, direction, and flow dynamics of the septal defect is essential when planning support. Standard VA-ECMO increases afterload, potentially raising ventricular filling pressures and exacerbating shunting across the defect. This can worsen hypoxemia unless additional venting or alternative strategies are employed. In selected cases, the addition of an Impella pump to VA-ECMO (known as the “Ecpella” configuration) can mitigate these effects by providing targeted LV unloading. Alternatively, left atrial VA-ECMO configurations have been reported to achieve effective decompression and improved systemic oxygenation.^20^ Conversely, a stand-alone Impella device without ECMO support may inadvertently intensify right-to-left shunting through a VSD, precipitating systemic desaturation.^21^

**Figure 2:**
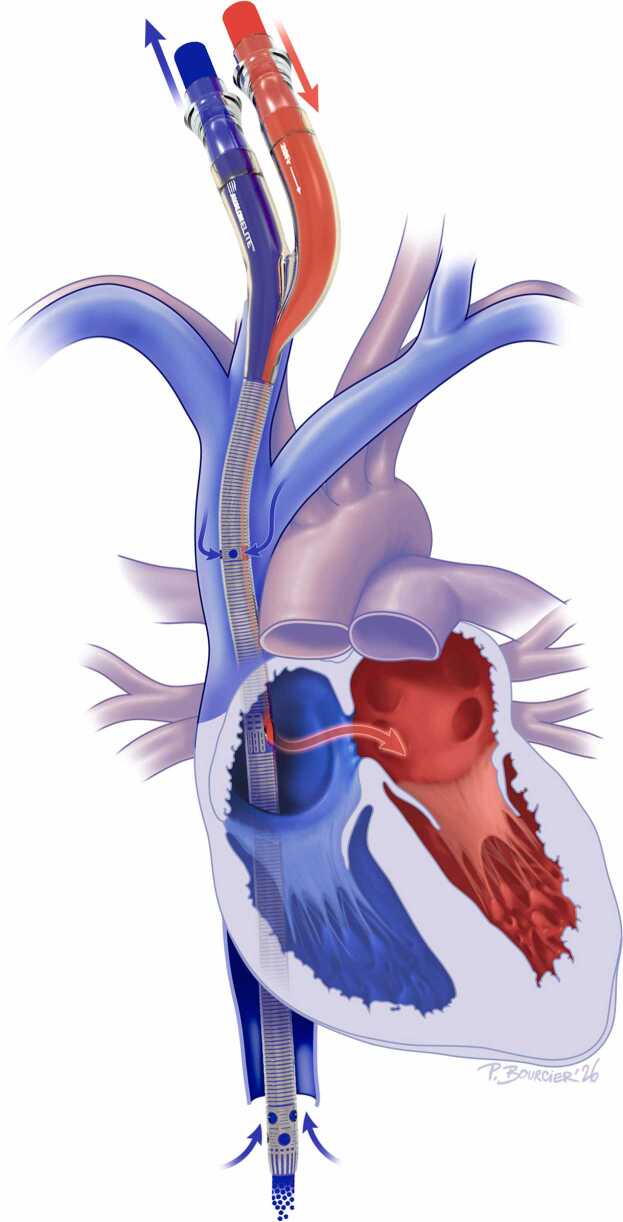
Bicaval, dual-lumen venovenous ECMO configuration in the setting of an ASD and Eisenmenger syndrome. Reinfused, oxygenated blood from the ECMO circuit (red arrows) is directed through the ASD toward the left atrium. ECMO, extracorporeal membrane oxygenation; ASD, atrial septal defect.

An innovative approach in patients with a more sustained ventricular function involves leveraging the Eisenmenger physiology itself. By positioning a Crescent or Avalon double-lumen cannula such that the return jet is directed across the ASD, clinicians can create an oxygenated right-to-left shunt (Figure 2). This maneuver improves systemic oxygenation, reduces pulmonary arterial pressures, and facilitates RV unloading.^22^

One should emphasize that the decision to initiate ECMO in a patient with Eisenmenger’s syndrome must also rely on a careful assessment of the bleeding risk during HLTx. Indeed, the combination of markedly hypertrophied systemic collateral vascularization arising from the pleura and mediastinum, together with the inflammatory and coagulopathic effects of ECMO, may raise the perioperative bleeding risk of HLTx to an unacceptable level. Therefore, the decision to initiate ECMO must take this potential additional risk into account and should be made only after thorough multidisciplinary discussion among the clinicians involved in the patient’s care.

## Conclusion

Bridging to HLTx can be challenging. While ECMO and mechanical circulatory support technologies have expanded the therapeutic window, optimal strategies require nuanced understanding of hemodynamics, oxygenation dynamics, and ventricular interaction.

No single configuration is universally superior, and success depends on timely initiation, precise cannulation strategy, and constant monitoring.

Future studies are needed to define standardized bridging protocols, optimize patient selection, and clarify long-term outcomes following HLTx supported by mechanical devices. Until then, individualized, physiology-guided approaches remain the cornerstone of effective bridging.

## Conflicts of Interest statement

The authors declare the following financial interests/personal relationships, which may be considered as potential competing interests: Olaf Mercier reports was provided by Marie-Lannelongue Hospital. If there are other authors, they declare that they have no known competing financial interests or personal relationships that could have appeared to influence the work reported in this paper.
